# The Adolescent Cardio-Renal Intervention Trial (AdDIT): retinal vascular geometry and renal function in adolescents with type 1 diabetes

**DOI:** 10.1007/s00125-017-4538-2

**Published:** 2018-02-02

**Authors:** Paul Z. Benitez-Aguirre, Tien Y. Wong, Maria E. Craig, Elizabeth A. Davis, Andrew Cotterill, Jennifer J. Couper, Fergus J. Cameron, Farid H. Mahmud, Tim W. Jones, Lauren A. B. Hodgson, R. Neil Dalton, David B. Dunger, Kim C. Donaghue, The Adolescent Type 1 Diabetes Cardio-Renal Intervention Trial (AdDIT), Sally Marshall, Jane Armitage, Polly Bingley, William Van’t Hoff, David Dunger, R. Neil Dalton, Denis Daneman, Andrew Neil, John Deanfield, Tim Jones, Kim Donaghue

**Affiliations:** 1 Institute of Endocrinology and Diabetes, The Children’s Hospital at Westmead, 170 Hawkesbury Rd, Locked Bag 4001, Westmead, NSW 2145 Australia; 2 Discipline of Child and Adolescent Health, University of Sydney, Sydney, NSW Australia; 3 Centre for Eye Research Australia, Melbourne, VIC Australia; 4 Singapore Eye Research Institute, Singapore National Eye Centre, Singapore, Republic of Singapore; 5 Duke-NUS Medical School, National University of Singapore, Singapore, Republic of Singapore; 6 School of Women’s and Children’s Health, University of New South Wales, Sydney, NSW Australia; 7 Department of Endocrinology and Diabetes, Princess Margaret Hospital for Children, Perth, WA Australia; 8 Telethon Kids Institute, University of Western Australia, Perth, WA Australia; 9 University of Queensland, Brisbane, QLD Australia; 10 Endocrinology and Diabetes Centre, Women’s and Children’s Hospital, Adelaide, SA Australia; 11 Robinson Research Institute, University of Adelaide, Adelaide, SA Australia; 12 Department of Endocrinology and Diabetes, Royal Children’s Hospital, Melbourne, VIC Australia; 13 Murdoch Children’s Research Institute, Melbourne, VIC Australia; 14 The University of Melbourne, Melbourne, VIC Australia; 15 Division of Endocrinology, Hospital for Sick Children, Toronto, ON Canada; 16 WellChild Laboratory, St Thomas’ Hospital, King’s College London, London, UK; 17 Department of Paediatrics, University of Cambridge, Box 116, Level 8, Cambridge Biomedical Campus, Cambridge, CB2 0QQ UK

**Keywords:** AdDIT, Adolescents, Diabetic retinopathy, Microvascular complications, Nephropathy, Retinal vascular geometry, Type 1 diabetes

## Abstract

**Aims/hypothesis:**

We examined the hypothesis that elevation in urinary albumin creatinine ratio (ACR) in adolescents with type 1 diabetes is associated with abnormal retinal vascular geometry (RVG) phenotypes.

**Methods:**

A cross-sectional study at baseline of the relationship between ACR within the normoalbuminuric range and RVG in 963 adolescents aged 14.4 ± 1.6 years with type 1 diabetes (median duration 6.5 years) screened for participation in AdDIT. A validated algorithm was used to categorise log_10_ ACR into tertiles: upper tertile ACR was defined as ‘high-risk’ for future albuminuria and the lower two tertiles were deemed ‘low-risk’. RVG analysis, using a semi-automated computer program, determined retinal vascular calibres (standard and extended zones) and tortuosity. RVG measures were analysed continuously and categorically (in quintiles: Q1–Q5) for associations with log_10_ ACR and ACR risk groups.

**Results:**

Greater log_10_ ACR was associated with narrower vessel calibres and greater tortuosity. The high-risk group was more likely to have extended zone vessel calibres in the lowest quintile (arteriolar Q1 vs Q2–Q5: OR 1.67 [95% CI 1.17, 2.38] and venular OR 1.39 [0.98, 1.99]) and tortuosity in the highest quintile (Q5 vs Q1–Q4: arteriolar OR 2.05 [1.44, 2.92] and venular OR 2.38 [1.67, 3.40]). The effects of retinal vascular calibres and tortuosity were additive such that the participants with the narrowest and most tortuous vessels were more likely to be in the high-risk group (OR 3.32 [1.84, 5.96]). These effects were independent of duration, blood pressure, BMI and blood glucose control.

**Conclusions/interpretation:**

Higher ACR in adolescents is associated with narrower and more tortuous retinal vessels. Therefore, RVG phenotypes may serve to identify populations at high risk of diabetes complications during adolescence and well before onset of clinical diabetes complications.

## Introduction

Despite significant advances in the management of type 1 diabetes, premature cardiovascular mortality has not been significantly ameliorated, with most of the increase in cardiovascular risk explained by diabetic nephropathy [1]. An effective method of identifying individuals who will develop severe microvascular complications, including diabetic nephropathy, remains elusive. While urinary albumin excretion measurement, a functional measure, remains the favoured method for identifying and monitoring persons who develop diabetic nephropathy, it is clear that not all individuals develop albuminuria or proteinuria before progressing to end-stage renal disease [2–4]. Furthermore, persons diagnosed with diabetes during childhood may have well over a decade of diabetes duration before transition to adult care. However, children and adolescents do not typically receive treatment during this prolonged period, as clinically overt complications are infrequent and hypertension uncommon. Nonetheless, evidence suggests that growth and puberty are critical periods that accelerate the systemic endotheliopathy leading to microvascular complications [5, 6].

The retina offers a unique opportunity to visualise and study the vasculature (especially the microvasculature) in vivo. Previous retinal vascular geometry (RVG) studies considered the proximal branches of central retinal vessels only [7]. We recently demonstrated the adverse effect of glycaemic burden on more peripheral retinal vessel calibres in youth with type 1 diabetes [8] and postulate that the earliest adverse phenotypes will be apparent in the peripheral retina. In adult studies of cardiovascular disease, arteriolar narrowing has been associated with higher blood pressure and greater cardiovascular risk, while venular changes have been separately associated with proteinuria.

Evidence suggests an inherent predisposition to the development of diabetes micro- and macrovascular complications in a proportion of the population [9, 10]. Identifying persons at high risk at the earliest possible time may allow for a preventive intervention strategy ideally during early life. There is, however, a paucity of data in paediatric populations with type 1 diabetes prior to onset of clinical diabetes complications, arguably a period when the greatest benefit from intervention may be derived and complications prevented rather than ameliorated. The Adolescent Type 1 Diabetes Cardio-Renal Intervention Trial (AdDIT; http://isrctn.com registration number 91419926) is the first interventional study in a group of normoalbuminuric adolescents with type 1 diabetes determined to be at high risk of developing kidney disease based on their urinary albumin creatinine ratio (ACR) [11].

In this study, we examine the cross-sectional associations at baseline between urinary ACR and RVG measures in adolescents with type 1 diabetes screened for participation in AdDIT. We hypothesised that higher urinary ACR is associated with an adverse RVG phenotype involving both arterioles and venules in the peripheral retina.

## Methods

### Study population

A cross-sectional study was carried out at baseline of a multinational cohort comprising 1041 normoalbuminuric adolescents with type 1 diabetes, screened for eligibility to the AdDIT, with mean age 14.4 years, median diabetes duration 6.5 years and HbA_1c_ 69 mmol/mol (8.5%). Data for the current study were collected at the study baseline visit prior to, or within 6 months of, trial commencement. We present the baseline data only. Treatment outcomes and unmasking of treatment allocation are beyond the scope of this paper. Briefly, in the multicentre trial across the UK, Australia and Canada, adolescents considered to be at ‘high-risk’ of diabetic nephropathy were randomised to an ACE inhibitor (ACE-I) and/or statin (HMG-CoA reductase inhibitor) or placebo [12], while the ‘low-risk’ cohort was followed as a natural history observational cohort.

This study conformed to the Declaration of Helsinki and was approved by The Cambridge University Research Ethics Committee and the local ethics committees of each participating centre. Written informed consent was obtained from the parents and assent from all study participants.

### Retinal photography and measurements

**Fig. 1:**
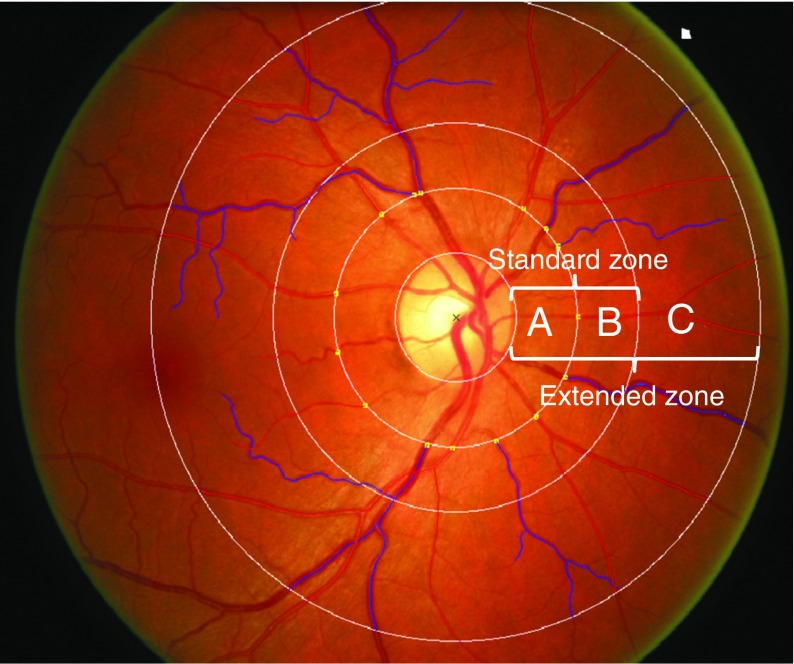
Retinal photograph taken at baseline, showing central optic disc field and macula field. Retinal zones according to SIVA: standard zone (A+B), extended zone (A + B + C)

Of the 1041 participants, 982 (94%) had full clinical data and standardised digital two-field retinal photographs from both eyes taken at baseline (central optic disc field and macula field). There were no clinically or biochemically significant differences between those with and without retinal photographs. We excluded those with ungradable photographs due to either poor quality of retinal photographs (*n* = 14, 1.4%) or for whom fewer than four of the largest vessels could be traced by the computer-assisted program in either eye (*n* = 5, 0.5%). Thus, 963 (92%) participants were included in analyses for this report. RVG was assessed using SIVA (Singapore I Vessel Assessment, Singapore, Republic of Singapore), a semi-automated computer-assisted image program. A single trained grader, masked to participants’ identities, applied the program to each retinal photograph, to measure retinal microvascular geometric variables within a concentric zone between the optic disc margin and two optic disc diameters away from the optic disc margin. The grader allowed the software to detect the centre of the optic disc and divided the region into three subzones surrounding the optic disc, zones A, B, C, corresponding to 0.5, 1.0 and 2.0 optic disc diameters away from the optic disc margin, respectively (Fig. 1). Once the optic disc and the three concentric subzones were appropriately located, the grader executed the program to trace all vessels. The grader ensured that all arterioles and venules were correctly identified. The software combined the individual measurements into summary indices of vessel calibres: the six largest vessels were used to calculate the central retinal arteriolar equivalent (CRAE) and central retinal venular equivalent (CRVE) in the standard zone B; the mean width of all measurable arterioles in the extended zone (exMWa) and mean width of all measurable venules in the extended zone (exMWv) (out to zone C) were calculated and arteriolar curvature tortuosity (CTa) and venular curvature tortuosity (CTv) were calculated as measures of vessel undulation, as previously described [13].

### Renal function measures

Centralised assessment of all urine samples was performed at The WellChild laboratory at The Evelina Children’s Hospital, London, UK. Samples were stored at −70°C prior to shipping. Urine albumin was measured using laser immunonephelometry (Siemens BN Prospec; Siemens Healthcare, Erlangen, Germany) and, for concentrations <2.1 mg/l, by an ELISA [11]. Urine creatinine was measured using a chromatographic stable isotope dilution electrospray MS–MS method. For each participant, the two time point log_10_ ACR measures, each based on three consecutive early morning samples at two separate visits, were averaged on the log_10_ ACR scale and the average residual calculated using age, sex and duration and the coefficients from the previously described linear regression model in the Oxford Regional Prospective Study (ORPS) cohort [14]. High-risk ACR was defined as being in the highest tertile, within the normoalbuminuric range, according to a standardised ACR that predicted 85% of adolescents with type 1 diabetes who later developed microalbuminuria and 100% who developed clinical proteinuria in the ORPS [15]. The upper ACR tertile (high-risk group) was assigned to residual value >1.2, the middle ACR tertile to values of 0.8–1.2 and lower ACR tertile to values <0.8. For this study, the lower two tertiles were combined for analysis as the low-risk group [11].

The eGFR was calculated using the following formula: eGFR (ml min^−1^ 1.73 m^−2^) = 42 × height (cm) / plasma creatinine (μmol/l) [12]. Hyperfiltration was defined as eGFR >135 ml min^−1^ 1.73 m^−2^ [16].

### Other variables

HbA_1c_ was analysed locally at each centre, using DCCT aligned methods (HbA_1c_ Variant analyser [Bio-Rad Laboratories, Hercules, CA, USA], Adams Arkray [Kyoto, Japan], Vantage analyser [Siemens Diagnostics, Camberley, UK] or DCA 2000, [Siemens Diagnostics, Tarrytown, NY, USA]). Lipid profile measurements (cholesterol, HDL-cholesterol, LDL-cholesterol, triacylglycerol) were measured using routine laboratory methods.

The standard deviation score (SDS) was calculated for height, weight and BMI according to the least-mean squares method [17]. Blood pressure was measured using an Omran M6 blood pressure monitor (Hoofddorp, the Netherlands) and/or Dinamap monitor (Tampa, FL, USA) using an appropriate sized cuff. Age- and sex-related percentiles and SDS for systolic blood pressure (SBP) and diastolic blood pressure (DBP) were calculated according to published standards [18].

This study was approved by the Human Research Ethics Committees of each participating centre. Informed consent was obtained from participants and their families.

### Statistics

Descriptive data are summarised as mean ± SD for parametric data and median (interquartile range [IQR]) for non-parametric data. Urinary ACR was log_10_ transformed for analysis and ACR groups were expressed as categorical variables (high-risk [upper tertile] vs low-risk [the two lower tertiles]). Thresholds were explored and quintiles deemed the optimal category for analysis. RVG measures were analysed as both continuous and categorical variables (quintiles). Mean differences in retinal vascular measures between high-risk and low-risk groups were determined using independent samples *t* tests for normally distributed variables or Mann–Whitney *U* tests for non-parametric variables. A χ^2^ test was used to compare the likelihood of being in the high-risk ACR group depending on retinal vascular measure quintiles.

Associations between log_10_ ACR, log_10_ eGFR and retinal vascular measures as continuous variables were examined using linear regression models. Binary logistic regression was used to explore threshold effects of retinal vascular measures, arriving at quintiles as the optimal grouping as predictors of high-risk and low-risk ACR groups. For vessel calibres, Q2–Q5 were combined into a single category for analysis. For tortuosity Q1–Q4, which were not statistically significantly different, were combined into a single category for analysis. Univariable regression analyses were carried out and multivariable regression models built using variables that were significant in univariable analysis, including HbA_1c_, BMI, SBP, lipids and duration. To test our proposed hypotheses that both arteriolar and venular components of the microvascular system contribute to the risk of microvascular dysfunction, we created two compound binary variables: (1) exMWaQ1 & CTvQ5 (the lowest exMWa quintiles and highest CTv quintile) and (2) exMWaQ1 & exMWvQ1 (the lowest quintiles of exMWa and exMWv). All statistical analyses were conducted using IBM SPSS version 22, Armonk, NY, USA.

## Results

### Participant characteristics

**Fig. 2:**
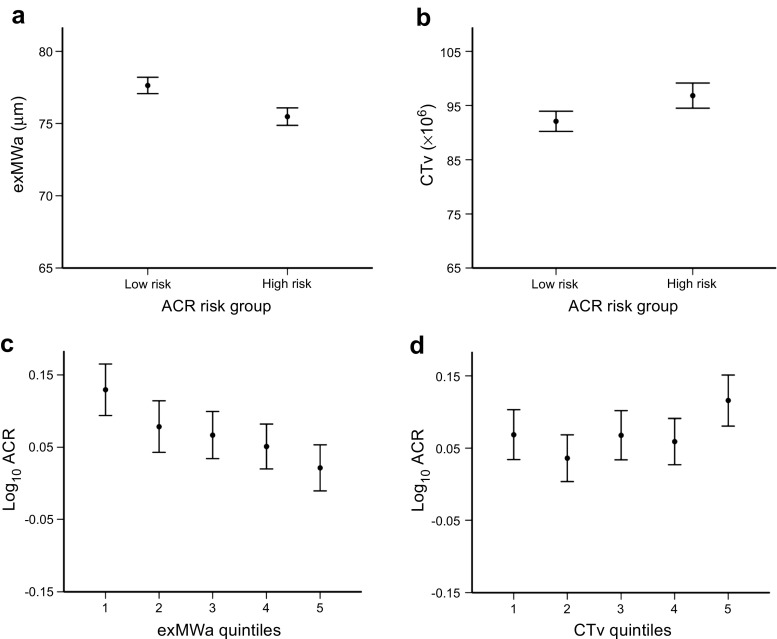
Associations between RVG measures and ACR. (**a**, **b**) RVG measures by ACR risk groups. Retinal arteriolar calibre in the extended zone was narrower (**a**, *p*<0.001) and tortuosity of venules was greater (**b**, *p*=0.005) in the high-risk group than in the low-risk group. (**c**, **d**) Continuous association between log_10_ ACR by RVG measure quintiles. Data for all panels are presented as mean and 95% CIs. *p* values represent ANOVA for whole-group analysis. *p*<0.001 in (**c**); *p*=0.02 in (**d**)

Characteristics of participants in the high-risk and low-risk groups are shown in Table 1. Adolescents in the high-risk group were younger, with shorter diabetes duration, lower BMI, higher SBP SDS and higher eGFR than those in the low-risk group. There were no significant differences in sex distribution, HbA_1c_ or lipid profiles between the two groups. Participants in the high-risk ACR group had narrower arteriolar and venular calibres in both standard (CRAE, CRVE) and extended zones (exMWa, exMWv) (Fig. 2) as well as more tortuous vessels (CTa and CTv) compared with those in the low-risk group.

**Table 1:** Clinical characteristics and retinal vascular measures by ACR risk group

Characteristic	Low-risk (*n*=517)	High-risk (*n*=446)	*p* value
Male sex, *n* (%)	276 (53)	237 (53)	0.9
Age, years	14.5 ± 1.6	14.2 ± 1.6	0.001
Diabetes duration, years	7.4 (4.9–10.2)	5.4 (3.6–8.1)	<0.001
Height, cm	165.0 ± 10.4	163.6 ± 10.6	0.049
Weight, kg	61.8 ± 13.6	58.4 ± 13.4	<0.001
BMI, kg/m^2^	21.9 (19.7–24.6)	20.9 (18.8–23.5)	<0.001
BMI SDS	0.95 ± 1.00	0.72 ± 1.05	0.001
Waist, cm	76.0 ± 10.0	74.3 ± 9.2	0.01
Waist:height	0.46 ± 0.06	0.45 ± 0.05	0.07
SBP, mmHg	115.6 ± 11.3	116.5 ± 12.0	0.3
SBP SDS	−0.14 ± 1.05	0.03 ± 1.13	0.02
DBP, mmHg	65.3 ± 8.1	66.1 ± 8.1	0.2
DBP SDS	0.90 ± 0.92	1.01 ± 0.93	0.1
Smoking, proportion (%)	5/522 (1.0)	3/399 (0.8)	0.6
HbA_1c_, mmol/mol	68 ± 14	69 ± 16	0.3
HbA_1c_, %	8.4 ± 1.3	8.5 ± 1.4	0.3
Cholesterol, mmol/l	4.40 ± 0.86	4.38 ± 0.84	0.7
eGFR, ml min^−1^ 1.73 m^−2^	122 ± 21	129 ± 24	<0.001
RVG			
CRAE, μm	154.2 ± 12.3	151.7 ± 12.3	0.002
CRVE, μm	217.8 ± 17.5	215.6 ± 17.7	0.045
exMWa, μm	77.6 ± 6.6	75.5 ± 6.6	<0.001
exMWv, μm	88.8 ± 7.4	87.0 ± 7.9	0.001
CTa ×10^6^	96.1 ± 1.3	99.7 ± 1.4	0.035
CTv ×10^6^	89.8 ± 1.2	93.7 ± 1.3	0.006

Data are presented as mean ± SD for parametric data, median (IQR) for non-parametric data and *n* (%) or proportion (%) for discrete data

### Linear regression

In univariable analysis, log_10_ ACR was inversely associated with BMI SDS (β −0.024 [95% CI −0.038, −0.009], *p* = 0.002) and diabetes duration (β −0.017 [95% CI −0.021, −0.012], *p* < 0.001) but not with HbA_1c_ (β 0.0004 [95% CI −0.0006, 0.0014], *p* = 0.5), SBP SDS (β 0.010 [95% CI −0.004, 0.024], *p* = 0.1) or cholesterol (β −0.009 [95% CI −0.027, 0.008], *p* = 0.3).

Log_10_ ACR was inversely associated with the calibres of the six largest arterioles (CRAE) in extended zones (exMWa and exMWv) but not the calibres of the six largest venules (CRVE). The compound variables (exMWaQ1 & CTvQ5; exMWaQ1 & exMWvQ1) showed an additive effect on log_10_ ACR (Table 2).

**Table 2:** RVG measures are associated with urinary albumin excretion

RVG measure	Linear regression: outcome log_10_ ACR				Logistic regression: outcome high-risk ACR group			
	Univariable model		Multivariable model^a^		Univariate model		Multivariable model^b^	
	β (95% CI)	*p* value	β (95% CI)	*p* value	OR (95% CI)	*p* value	OR (95% CI)	*p* value
RVG calibre^c^								
CRAE (μm)	−0.002 (−0.003, −0.001)	0.004	−0.001 (−0.002, 0.0003)	0.1	1.20 (0.89, 1.65)	0.3	1.04 (0.73, 1.49)	0.8
CRVE (μm)	−0.001 (−0.001, 0.0003)	0.2	0.0001 (−0.001, 0.001)	0.8	1.27 (0.92, 1.74)	0.1	0.99 (0.70, 1.41)	0.95
exMWa (μm)	−0.006 (−0.008, −0.003)	<0.001	−0.004 (−0.006, −0.002)	0.001	1.96 (1.42, 2.71)	<0.001	1.67 (1.17, 2.38)	0.005
exMWv (μm)	−0.003 (−0.004, −0.001)	0.01	−0.001 (−0.003, 0.0008)	0.3	1.77 (1.29, 2.44)	<0.001	1.39 (0.98, 1.99)	0.07
RVG tortuosity^d^								
Log_e_ CTa ×10^6^	0.048 (−0.007, 0.103)	0.09	0.079 (0.02, 0.13)	0.005	1.78 (1.30, 2.46)	<0.001	2.05 (1.44, 2.92)	<0.001
Log_e_ CTv ×10^6^	0.067 (0.004, 0.13)	0.03	0.096 (0.03, 0.16)	0.003	2.15 (1.56, 2.98)	<0.001	2.38 (1.67, 3.40)	<0.001
Combined RVG models								
exMWaQ1 & CTvQ5 vs others^e^	0.12 (0.06, 0.17)	<0.001	0.11 (0.05, 0.17)	<0.001	3.42 (1.96, 5.97)	<0.001	3.32 (1.84, 5.96)	<0.001
exMWaQ1 & exMWvQ1 vs others^f^	0.08 (0.03, 0.13)	0.002	0.05 (−0.01, 0.10)	0.09	2.24 (1.43, 3.50)	<0.001	1.77 (1.08, 2.90)	0.02

Each row represents a separate model. Outcome variable for linear regression modelling was log_10_ ACR. HbA_1c_, sex, lipids and SBP SDS were not significant in univariable models. Outcome variable for logistic regression modelling was high-risk ACR group. HbA_1c_, sex and lipids were not significant in univariable models

^a^Multivariable models are adjusted for BMI SDS and duration

^b^Multivariable models are adjusted for BMI SDS, duration and SBP SDS

^c^Logistic regression outcome is shown for Q1 vs Q2–Q5

^d^Logistic regression outcome is shown for Q5 vs Q1–Q4

^e^Logistic regression outcome is shown for exMWaQ1 & CTvQ5 vs the others

^f^Logistic regression outcome is shown for exMWaQ1 & exMWvQ1 vs the others

In multivariable models for each RVG variable, log_10_ ACR was associated with narrower arteriolar calibres across extended zones (exMWa) and greater arteriolar and venular tortuosity (CTa and CTv). There was a similar trend for venular calibres (exMWv) but this did not reach statistical significance. The compound variable exMWaQ1 & CTvQ5 remained significant with an additive effect on log_10_ ACR. The associations remained significant after adjusting for BMI SDS and diabetes duration. The compound variable exMWaQ1 & exMWvQ1 showed a similar trend but did not reach statistical significance (Table 2).

### Logistic regression

In univariable analysis, high-risk ACR was associated with shorter diabetes duration (OR 0.87 [95% CI 0.84, 0.91], *p* < 0.001), younger age (OR 0.87 [95% CI 0.80, 0.94], *p* = 0.001), lower BMI SDS (OR 0.80 [95% CI 0.70, 0.91], *p* = 0.001) and higher SBP SDS (OR 1.14 [95% CI 1.01, 1.29], *p* < 0.001), while its association with HbA_1c_ (OR 1.005 [95% CI 0.996, 1.014], *p* = 0.2) was not significant.

In multivariable analysis, high-risk ACR was associated with the lowest quintile for calibres in the extended zone (exMWa and exMWv, Q1 vs Q2–Q5) and the highest quintile for vessel tortuosity (CTa and CTv, Q5 vs Q1–Q4). The compound variables (exMWaQ1 & CTvQ5; exMWaQ1 & exMWvQ1) showed an additive effect for high-risk ACR (Table 2). These findings remained significant after adjusting for BMI SDS, duration and blood pressure SDS.

eGFR was not significantly associated with RVG variables, either continuously or categorically. No associations were found between hyperfiltration and RVG.

## Discussion

In this study we demonstrate that higher urinary ACR in adolescents with type 1 diabetes is associated with a specific RVG phenotype even before onset of clinical complications. Higher ACR was associated with narrower retinal vascular calibres and increased vessel tortuosity even within the normoalbuminuric range.

Our study adds significant new knowledge to this field. First, we provide detailed and extensive analysis over a wider area of the retina (extended zone), measuring not only vessel calibres but also vessel tortuosity. Second, we report that extended zone calibres, both arteriolar and venular (exMWa and exMWv, respectively) rather than CRAE and CRVE, were more robust predictors of log_10_ ACR. Narrower retinal arterioles (CRAE) in the standard zone have previously been associated with vascular dysfunction and adverse cardiovascular events [19–21]; here, we describe a novel association with vessel calibres in the extended zone. In our study, participants with the narrowest retinal calibres in the extended zone were twice as likely to be in the high-risk ACR group. This pattern suggests that inappropriate microvascular vasoconstriction occurs in the high-risk ACR group, compounding the neuroretinal ischaemia and pro-inflammatory state present in the diabetes milieu early in the disease process. Since the microvasculature provides the greatest contribution to systemic vascular resistance, we postulate that persistent inappropriate arteriolar vasoconstriction will in due course result in systemic blood pressure elevation [21] contributing to shear-stress on the vessel wall and accelerating microvascular pathology. This is in keeping with our observation that the high-risk group had higher SBP SDS, albeit within the normal range. Narrower venules in the setting of inadequate perfusion are also likely to represent dysfunctional autoregulation, which together with higher systemic blood pressure may increase hydrostatic vessel damage. There are clear mechanistic prospects such as hyperglycaemia leading to a downregulation of Ca^2+^-activated K^+^ (BK) channels that are important in vasodilatation [22]. Such risk may be mediated through inherited individual differences in sensitivity and response to similar glycaemic exposure [23].

We also demonstrate that greater tortuosity was associated with higher log_10_ ACR. Notably, the highest quintile of venular tortuosity was associated with greatest probability of being in the high-risk ACR group. We previously demonstrated that greater tortuosity was associated with increased risk of retinopathy [24] and renal dysfunction in our clinic population [25, 26]. We now present the first multinational cohort supporting these findings. We speculate that increased tortuosity may be the result of a maladaptive compensatory increase in vessel density (or early neovascularisation), to improve neuroretinal perfusion, in a predisposed population.

Third, we provide evidence supporting a unifying haemodynamic model integrating afferent (arteriolar) and efferent (venular) components of the microcirculation whereby both components contribute to risk in an additive manner. We report an adverse retinal phenotype comprising narrower vessel calibres and greater tortuousity that was associated with a significantly greater likelihood of a person being in the high-risk ACR group than when either of these measures were used alone.

We observed that individuals in the high-risk ACR group were younger, had shorter diabetes duration and higher blood pressure but no difference in glycaemic control compared with the low-risk group. Thus, our data suggest the high-risk ACR group, screened through ACR, may indeed be a population predisposed to earlier onset of complications and likely to benefit from earlier intervention. Current evidence supports both genetic and metabolic mechanisms that protect against and predispose to diabetes complications [9, 10, 27]. However, a clinically measurable and reproducible biomarker associated with such risk has been elusive. The Renin Angiotensin System Study (RASS), which examined young adults with type 1 diabetes and normoalbuminuria, found that central retinal vascular calibres were associated with histological renal glomerular indices and their progression [7]. This was observed despite there being no functional differences in albumin excretion between the intervention (renin–angiotensin system blockade) and placebo groups, thus highlighting the relevance of the retinal microvasculature as an early biomarker of diabetic nephropathy. Retinal vascular phenotypes may reflect such genetic and metabolic predispositions and thus may serve to identify persons at high risk of future complications. In addition, retinal vascular phenotypes in the extended zones may be more responsive to intervention, including ACE-I and statin therapy and more intensive glycaemic control, early in the disease process.

The strengths of our study include the multinational collaboration and large sample size, standardised retinal photography images and single-centre grading of retinal images masked to clinical and biochemical participant data. However, these are the baseline data and therefore cross-sectional in nature. The longitudinal follow-up from AdDIT will provide invaluable insights into the mechanisms of diabetes complications and potential benefits of early ‘pre-complications’ intervention in paediatric cohorts. In addition to their role as potential biomarkers for risk stratification of future diabetes complications, RVG measures may provide an opportunity to quantify the effect of intervention on the microvasculature in vivo. Furthermore, these RVG quantification techniques may be applied to other previously conducted interventional study cohorts such as RASS and the DCCT. Long-term follow-up of such cohorts may determine the potential to shift the current screening paradigm from one of detection and treatment of diabetes complications to one of identification of persons at risk and prevention of microvascular complications.

In conclusion, we demonstrate that urinary ACR is associated with RVG variables prior to the onset of clinical complications in adolescents with type 1 diabetes. Higher ACR and greater likelihood of being in the high-risk ACR group were associated with narrower and more tortuous retinal vessels, particularly in the extended zones. RVG may assist in identifying individuals at high risk of complications early in the disease process and may provide useful biomarkers by which to quantify response to diabetes therapy.

## Data Availability

The datasets generated during and/or analysed during the current study are not publicly available due to data currently being under embargo for publication but are available from the corresponding author on reasonable request.

## Appendix

AdDIT investigators in Australia: Tim Jones (Princess Margaret Hospital for Children and University of Western Australia, Perth, WA, Australia), Kim Donaghue (The Children’s Hospital at Westmead and University of Sydney, Sydney, NSW, Australia), Maria Craig (The Children’s Hospital at Westmead, University of Sydney, and University of New South Wales, Sydney, NSW, Australia), Paul Benitez-Aguirre (The Children’s Hospital at Westmead and University of Sydney, Sydney, NSW, Australia), Fergus Cameron (Royal Children’s Hospital, Murdoch Children’s Research Institute and The University of Melbourne, Melbourne, VIC, Australia), Jennifer Couper (Women’s and Children’s Hospital and University of Adelaide, Adelaide, SA, Australia), Elizabeth Davis (Princess Margaret Hospital for Children and University of Western Australia, Perth, WA, Australia), Andrew Cotterill (University of Queensland, Brisbane, QLD, Australia), Bruce King (University of Newcastle, Newcastle, NSW, Australia), Charles Verge (Sydney Children’s Hospital and University of New South Wales, Sydney, NSW, Australia), Phil Bergman (Monash Children’s Hospital, Clayton, VIC, Australia), Christine Rodda (University of Melbourne, Melbourne, VIC, Australia).

AdDIT investigators in the UK: M. Loredana Marcovecchio (University of Cambridge, Cambridge, UK), John Deanfield (University College London, London, UK), Scott Chiesa (University College London, London, UK), Carlo Acerini (Addenbrooke’s Hospital and University of Cambridge, Cambridge, UK), Fran Ackland (Northampton General Hospital, Northampton, UK), Binu Anand (West Suffolk Hospital, NHS Foundation Trust, Bury St Edmunds, UK), Tim Barrett (Birmingham Children’s Hospital and University of Birmingham, Birmingham, UK), Virginia Birrell (James Cook Hospital, South Tees Hospitals NHS Foundation Trust, Middlesbrough, UK), Fiona Campbell (Leeds General Infirmary, The Leeds Teaching Hospitals NHS Trust, Leeds, UK), Marietta Charakida (King’s College London, London, UK), Tim Cheetham (Royal Victoria Infirmary, The Newcastle upon Tyne Hospitals NHS Foundation Trust and Newcastle University, Newcastle, UK), Chris Cooper (Stepping Hill Hospital, Stockport NHS Foundation Trust, Stockport, UK), Ian Doughty (Royal Manchester Children’s Hospital, Manchester, UK), Atanu Dutta (Stoke Mandeville Hospital, Aylesbury, UK), Julie Edge (John Radcliffe Hospital, Oxford, UK), Alastair Gray (University of Oxford, Oxford, UK), Julian Hamilton-Shield (University of Bristol and University Hospitals Bristol NHS Foundation Trust, Bristol, UK), James Heywood (University of Cambridge, Cambridge, UK), Nicola Leech (Newcastle General Hospital, The Newcastle upon Tyne Hospitals NHS Foundation Trust and Newcastle University, Newcastle, UK), Nick Mann (Royal Berkshire Hospital, Reading, UK), Richard Parker (University of Cambridge, Cambridge, UK), Gerry Rayman (Ipswich Hospitals NHS Trust, Ipswich, UK), Jonathon Mark Robinson (Royal Albert Edward Infirmary, Wrightington, Wigan and Leigh NHS Foundation Trust, Wigan, UK), Michelle Russell-Taylor (Wycombe Hospital, Buckingham Healthcare NHS Trust, High Wycombe, UK), Vengudi Sankar (Royal Bolton Hospital, Bolton NHS Foundation Trust, Bolton, UK), Anne Smith (Northampton General Hospital, Northampton, UK), Nandu Thalange (Norfolk and Norwich University Hospitals NHS Foundation Trust, Norwich, UK), Mark Wilson (University of Cambridge, Cambridge, UK), Chandan Yaliwal (Royal Berkshire Hospital, Reading, UK).

AdDIT investigators in Canada: Farid Mahmud (The Hospital for Sick Children and University of Toronto, Toronto, ON, Canada); Cheril Clarson (London Health Sciences Centre and Western University, London, ON, Canada); Jacqueline Curtis (The Hospital for Sick Children and University of Toronto, Toronto, ON, Canada), Denis Daneman, (The Hospital for Sick Children and University of Toronto, Toronto. ON, Canada), Etienne Sochett (The Hospital for Sick Children and University of Toronto, Toronto, ON, Canada).

## Abbreviations

ACE-I: ACE inhibitor
ACR: Albumin creatinine ratio
AdDIT: Adolescent Type 1 Diabetes Cardio-Renal Intervention Trial
CRAE: Central retinal arteriolar equivalent
CRVE: Central retinal venular equivalent
CTa: Arteriolar curvature tortuosity
CTv: Venular curvature tortuosity
DBP: Diastolic blood pressure
exMWa: Mean width of all measurable arterioles in the extended zone
exMWv: Mean width of all measurable venules in the extended zone
IQR: Interquartile range
ORPS: Oxford Regional Prospective Study
RASS: Renin Angiotensin System Study
RVG: Retinal vascular geometry
SBP: Systolic blood pressure
SDS: Standard deviation score
